# Community-Engaged Qualitative Study on Supporting Transgender and Gender-Diverse Standardized Patients in Medical Simulation

**DOI:** 10.1007/s11606-025-09640-1

**Published:** 2025-06-24

**Authors:** Steven Allon, Andrew Kittleson, Pepper Heifner, David Schlundt, Kemberlee Bonnet, Arna Banerjee, Christopher Terndrup

**Affiliations:** 1 Department of Medicine, Vanderbilt University Medical Center, Nashville, TN USA; 2 Medical Scientist Training Program, Vanderbilt University School of Medicine, Nashville, TN USA; 3 Program for LGBTQ Health, Vanderbilt University Medical Center, Nashville, TN USA; 4 Department of Psychology, Vanderbilt University, Nashville, TN USA; 5 Vanderbilt University Medical Center, Nashville, TN USA

**Keywords:** Transgender healthcare, Medical simulation, Standardized patients, Health equity

## Abstract

**Background:**

Transgender and gender-diverse (TGD) individuals experience high rates of adverse physical and mental health outcomes, in part due to negative healthcare experiences that discourage healthcare utilization. Affirming practices by providers can mitigate this distress, but medical education to build these skills is limited. Medical simulation offers an opportunity to enhance affirming communication, but best practices in supporting TGD standardized patients (SPs) are lacking.

**Objective:**

This study sought to develop a preliminary guideline on recruitment, portrayal, and support of TGD SPs.

**Design:**

We utilized a modified Delphi technique to identify consensus guidelines for medical simulation programs incorporating TGD individuals as standardized actors in simulation scenarios.

**Participants:**

Fifty TGD community members and parents of TGD children in Middle Tennessee provided embodied knowledge.

**Approach:**

We administered a survey examining participants’ perspectives on recruitment, casting, and supporting TGD SPs in medical simulations. Thematic analysis was used to derive guideline statements, which were refined using participant feedback over two additional survey rounds.

**Key Results:**

Our sample consisted of 50 participants (96% TGD). We derived 44 guideline statements related to recruitment, casting, and supporting TGD SPs. Participants recommended recruitment of prospective TGD SPs through established venues holding trusted relationships with the TGD community, honestly conveying the benefits and drawbacks of this work. A gradient of acceptable casting decisions emerged, anchored by the gender identity of the simulated patient, to ensure shared lived experiences between an SP and their cast role. Participants offered strategies throughout simulation encounters to enhance TGD SPs’ agency, facilitate psychological safety, and process emotions to improve simulation programs’ support of TGD SPs.

**Conclusions:**

We utilized a modified Delphi technique with TGD community members to derive a consensus guideline to support TGD SPs. Our study addressed key unresolved questions in the literature, particularly around recruitment and casting, with a high level of agreement.

**Supplementary Information:**

The online version contains supplementary material available at 10.1007/s11606-025-09640-1.

## INTRODUCTION

Transgender and gender-diverse (TGD) individuals comprise an estimated 1.6% of the US adult population.^1^ TGD individuals experience adverse physical and mental health outcomes at significantly higher rates than cisgender individuals.^2^ The source of these inequities is multifactorial, driven in part by negative healthcare experiences that discourage healthcare utilization, decrease retention in care, and trigger patient distress.^3^ The provision of affirming care by well-trained providers can ameliorate distress.^4,5^

Educating future physicians on affirming care for TGD patients is a critical step to reversing health inequities. Although most US medical schools include LGBTQ+ topics, the curricula are typically limited to 11 h over 4 years, are primarily didactic, and rarely address transgender-specific experiences like transitioning.^6^ Clinical experience with TGD patients is also limited, evidenced by medical students’ low self-reported comfort and knowledge in caring for TGD patients.^7^ These curricular deficiencies threaten the preparedness and competency of future physicians in caring for this vulnerable population.

Medical simulation using standardized patients (SPs) portraying TGD patients offers a potential solution to this gap. Simulation allows medical trainees to gain experience interviewing TGD SPs in a psychologically safe environment.^8^ However, the lack of best practices for preparing and portraying TGD identities in medical simulation is a significant barrier. Only 50% of medical schools in the USA and Canada use TGD-related simulation cases, variably casting TGD SPs, cisgender SPs, and manikins to portray cases.^8^

A scoping review of TGD representation in simulation recommends collaboration with local communities to determine representation priorities.^9^ Key unresolved issues include casting decisions for SPs and strategies to support TGD SPs during simulation encounters. Previous studies have explored the perspectives of TGD SPs and healthcare providers, finding that TGD SPs bring authenticity to simulation encounters and may view the work as meaningful or even restorative. At the same time, they face emotional risks, particularly from microaggressions, which can be mitigated by a supportive simulation environment. Findings from these studies may have limited generalizability, however, as participants were affiliated with academic medical centers and may have been more willing to make educational concessions.^10,11^ To our knowledge, no prior study has examined the perspectives of TGD community members on supporting TGD SPs.

This study aimed to develop consensus guidelines for recruiting, casting, and supporting TGD SPs in medical simulation using feedback from TGD community members.

## METHODS

### Study Design and Oversight

We utilized a modified Delphi method, a structured and iterative consensus-building approach, to actively engage participants with critical expertise and generate guidelines based on their feedback.^12^ The study was approved by the Institutional Review Board at Vanderbilt University Medical Center (IRB #231463). All participants provided written informed consent.

### Participants

A purposive sample of prospective participants was recruited through the dissemination of a research flyer to institutional and community organizations that serve the needs of TGD adults in metropolitan Nashville, TN. Eligible participants were (1) community-dwelling adults who self-identified as transgender or gender-diverse or were parents of transgender or gender-diverse children, (2) aged 18 years or older, (3) literate in English, and (4) US citizens or permanent residents. Parents of TGD youth, regardless of gender identity, were included to offer proxy insights into the healthcare experiences of TGD minors often excluded from research. Individuals unable to give informed consent were excluded from the study. Participants received a $50 electronic Amazon gift card for completing each disseminated survey, with a maximum compensation of $150.

### Research Team

Our interdisciplinary team had expertise in TGD community engagement, medical simulation, TGD healthcare, and qualitative study design and analysis. This synergy provided unique insights into community recommendations, simulation program administration, and educational objectives. The team included both sexual and gender minority members and non-members, offering complementary perspectives on participant responses.

### Study Procedures

We conducted three rounds of surveys using a modified Delphi approach. Participants received a questionnaire via email each round. The Research Electronic Data Capture (REDCap) platform was utilized for study correspondence, data collection, and data storage.^13,14^

1. Round 1. The first-round questionnaire asked participants a series of open-ended questions about recruitment, casting, and support of TGD SPs. We derived questions from a review of primary literature of qualitative studies of TGD SPs and synthesis of scoping reviews on this topic,^8,9,10,11^ with review by two medical simulation program directors to ensure the topic review was comprehensive. Responses were coded with a hierarchically organized coding system, sorted by codes, and analyzed by the research team to identify common themes and generate a preliminary set of guideline statements.
2. Round 2. Participants reviewed and rated the proposed guideline statements from Round 1. For each item in the REDCap survey, participants were asked if they agreed or disagreed with the statement. For items they disagreed with, participants were prompted to suggest changes that would allow them to support the guideline. Data analysis involved quantitative assessment of the ratings (percent agreement) and qualitative analysis of the comments to identify potential revisions or additions to guideline statements. Items with at least 90% agreement were considered final and were not revised for reconsideration in the subsequent round.
3. Round 3. Participants reconsidered their initial ratings based on feedback from Round 2. They provided revised ratings and responses to resolve disagreements. Data analysis examined changes in ratings, identified opinion shifts, and determined the level of consensus for each item.

The final set of consensus guidelines was developed based on the aggregated ratings, qualitative feedback, and revisions from Round 3.

### Data Analysis

Round 1 data analysis was conducted using a thematic analysis approach. A codebook was collaboratively developed by four authors (S.A., A.K., D.S., and K.B.) using an iterative process that combined deductive and inductive approaches. Deductive codes were informed by the health systems science framework^15^ and clinical expertise, while inductive codes were generated through the review of a sample of participant responses. Participant responses were coded by two authors (S.A. and A.K.) (Supplementary Table 1). Initially, each coder independently coded 10%, discussed, and resolved discrepancies through consensus meetings. The remaining responses were split evenly and coded independently.

Themes were identified through consensus discussions among five authors (S.A., A.K., P.H., D.S., and K.B.). These themes were reviewed and refined through iterative discussions. Key themes were synthesized into guideline statements regarding the recruitment, casting, and support of TGD SPs. For each statement in the final round, at least 60% agreement was required for inclusion in the consensus guidelines. Statements with more than 75% agreement were categorized as strong recommendations, while those with 60–74% agreement were categorized as conditional recommendations. These thresholds align with median agreement levels reported in a systematic review of Delphi studies.^16^ To assess the impact of participant attrition, we conducted a worst-case sensitivity analysis assuming all nonresponders disagreed with each guideline statement.

## RESULTS

This study was conducted between December 1, 2023, and June 30, 2024. Seventy-three individuals were screened, and 69 (94.5%) were eligible to participate. Fifty participants provided complete responses to the first round, 39 (78%) to the second round, and 43 (86%) to the third round. Forty-eight participants were TGD (96%), and two participants (4%) were cisgender parents of a TGD child. Most participants (86%) were between ages 18 and 44 years old (Table 1).

**Table 1:** Baseline Characteristics of Participants (*N*=50)

Characteristic		*N* (%)
*Age*	18–24	9 (18%)
	25–34	26 (52%)
	35–44	8 (16%)
	45–54	5 (10%)
	55–64	1 (2%)
	65 or older	1 (2%)
Gender identity*	Transgender woman	8 (16%)
	Transgender man	13 (26%)
	Cisgender woman	2 (4%)
	Cisgender man	0 (0%)
	Non-binary	17 (34%)
	Genderqueer	3 (6%)
	Genderfluid	3 (6%)
	Agender	2 (4%)
	Two-Spirit	1 (2%)
	Other	1 (2%)
*Sex assigned at birth*	Female	11 (22%)
	Male	39 (78%)

^*^No participants identified as bigender or demigender

### Round 1 Themes

#### Recruitment

Participants stressed the importance of a positive relationship between the institution, its simulation program, and the TGD community. They recommended inclusive hiring processes that outline both the monetary and non-monetary benefits and acknowledge the emotional labor involved in SP work. Despite recruitment barriers, participants noted a pool of prospective TGD SPs exists and suggested active outreach by simulation programs. They also identified ethical recruitment sources and methods for TGD SPs.

#### Casting

Participants recommended that SPs should, whenever possible, share the gender identity of the simulated patient to ensure authentic representation, obtain feedback from SPs with lived experience, and avoid discrimination. Even with an “identical” alignment, consent from the TGD SP to engage in the simulation event is necessary for psychological safety.

**Figure 1:**
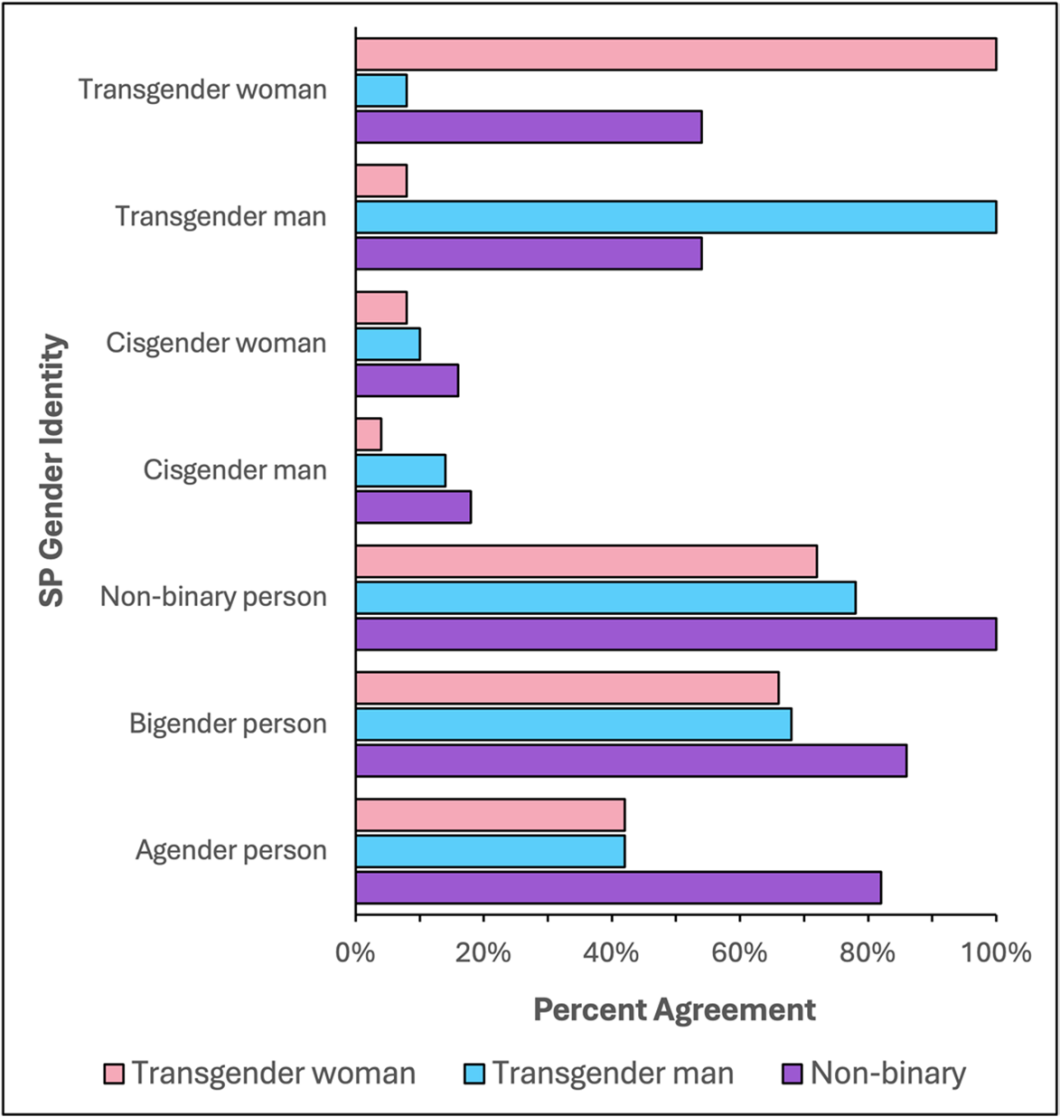
Quantitative responses on acceptable casting decisions based on a standardized actor’s gender identity to portray a simulated patient identifying as a transgender woman, transgender man, or non-binary individual (*SP*, standardized patient).

Participants provided insights into acceptable alternative casting decisions when an SP’s gender identity differed from that of the simulated patient (Fig. 1), emphasizing the importance of recognizing the diversity of individual experiences. They acknowledged that TGD individuals “may present in numerous ways depending on the context, safety, [and] experience of the individual’s own gender exploration.” One participant succinctly outlined consensus casting criteria: “Gender dysphoria must be experienced to really understand and react to questioning. Equally important is having experience in being a marginalized social group frequently under attack.”

##### Transgender Men and Women Portrayal

When an SP’s gender identity did not match the role, they recommended preferentially casting TGD individuals based on matching sex assigned at birth. For example, transfeminine individuals—a term inclusive of a range of gender identities of persons assigned male at birth (AMAB) who identify with a feminine gender experience, either wholly (usually transgender women) or in part—were preferred over TGD individuals assigned female at birth (AFAB) to portray a transgender woman as “they are more likely to understand [their] struggles and concerns.”

Cisgender representation was generally unacceptable due to concerns that the lack of lived experience could lead to “inaccuracies of reaction and demeanor” and stereotyped portrayals during simulation encounters. As one participant reflected, “Being trans is not a costume that someone can put on and take off at will.” Another participant drew a comparison to other minoritized groups, asking, “Do white persons portray Black persons? If so, they should not. There is a lived experience that cannot be trained.” However, participants were more accepting of this practice if the simulated patient’s gender identity matched the cisgender SP’s sex assigned at birth. For example, one participant noted that cisgender and transgender women “face many similar societal issues” that inform their portrayal. Moreover, “engaging in this labor may cause further harm to minoritized individuals, potentially necessitating the use of individuals from other backgrounds or identities.”

Participants strongly opposed casting transgender men as transgender women and vice versa, due to lack of lived experience and potential psychological harm, including worsening gender dysphoria.

##### Non-binary Portrayal

Recommendations were like those for transgender individuals. Importantly, participants indicated high acceptability of casting of transgender men, transgender women, bigender, and agender individuals. Bigender describes an individual who identifies with two genders, either at the same time or at different times, whereas agender describes individuals who do not identify with any gender.

##### Two-Spirit Portrayal

Two-Spirit is a culturally specific identity used by some Indigenous people in North America to describe a person who embodies both masculine and feminine spirits, or who holds a distinct gender or social role within their community. Participants emphasized that casting for Two-Spirit individuals should be considered separately due to intersectional considerations, cautioning that simulation programs “should be mindful of cultural appropriation.” They recommended restricting Two-Spirit roles to Two-Spirit SPs, with participants acknowledging their own lack of lived experience, as one participant indicated: “Could I, as a transgender woman, accurately portray a Two-Spirit person? I don’t think I could. I don’t [...] know what etiquette or pronouns a Two-Spirit person deems important.”

##### Cisgender Portrayal

Most participants recommended against casting TGD SPs as simulated patients who were explicitly identified as cisgender. This practice could harm SPs’ psychological safety and trigger gender dysphoria.

#### Case Modifications

Participants provided insights on how common medical simulation variations, such as manikins, videoconferencing software, and simulation case content, might affect appropriate portrayal. They advocated against altering casting decisions based on simulation modality. They also suggested best practices for affirming TGD SPs when voicing manikins and using videoconferencing software.

While most participants agreed that casting restrictions could be relaxed for simulations unrelated to gender-affirming care, a sizeable minority (26%) argued that all patient care for TGD SPs is inherently gender-affirming care and requires inclusive communication and respect. To illustrate their perspectives, nine participants referenced a scenario involving a TGD patient presenting with a broken arm. Some viewed this case as unrelated to gender-affirming care and appropriate for portrayal by a cisgender SP. As one participant noted, “You don’t need to know what [organs] I was born with to put my arm in a sling.” Others challenged this framing, invoking concerns related to “trans broken arm syndrome,” a term used in the medical literature to describe situations in which a transgender patient’s gender identity is inappropriately centered during unrelated clinical encounters.^17^ In this context, a cisgender SP “may not accurately identify where a learner is inappropriately focused on a trans identity in this scenario, as they may believe this is routinely how doctors may need to treat trans patients.” Finally, participants emphasized that even scenarios that appear neutral may involve gender-affirming care depending on the educator’s learning objectives: “If you need to train people how to treat a TGD individual who may be suffering a breakage that could be due to osteoporosis that may or may not be down to their estrogen medications being off[,] then it does matter.”

#### Support

Although the questionnaire included an item on optimal training for cisgender SPs portraying TGD simulated patients, participants expressed low acceptability for this practice. A qualitative evaluation of responses showed that 14% of participants firmly recommended against this practice, 24% conditionally recommended against it, and 62% conditionally recommended in favor of it, provided specific training and limitations were in place. Lack of acceptability was driven by several concerns, including an absence of lived experience among cisgender SPs, inauthentic and uninstructive experiences for trainees, and potential offensiveness to the TGD community. However, many participants also acknowledged the tension between ensuring authentic portrayals and protecting psychological safety. Cisgender representation, some noted, may “prevent potentially harmful labor” for TGD SPs, mitigating the emotional burden associated with repeated participation in simulations.

Given these mixed perspectives, we performed a thematic analysis of the suggested training for cisgender SPs but did not develop formal guideline statements. Key recommendations included mandatory cultural and clinical competency training, screening for transphobia, peer discussions or training by TGD community members, and recommendations against modifying an SP’s voice, appearance, or dress during encounters.

Participants made several recommendations to support TGD SPs before, during, and after simulation encounters. Before a simulation, they emphasized the importance of obtaining informed consent from TGD SPs for the specific case. They also recommended that trainees receive baseline TGD cultural competency training to reduce the risk of psychological harm. This training should cover respectful communication, including how to ask about chosen names and pronouns, basic terminology related to gender identity and expression, and common biases or microaggressions experienced by TGD individuals. Ideally, TGD voices should be included in the training. Participants also suggested informing trainees in advance that they will be working with TGD SPs to increase awareness and reduce microaggressions. During the simulation, they recommended measures to preserve TGD SPs’ agency and psychological safety, such as promising intervention by staff in unsafe situations, allowing optional breaks, and permitting external support members. After the simulation, they advised holding debrief sessions for TGD SPs to process emotions and provide feedback for program improvement as the “most valuable data on how to improve this process is going to come from people who have been through it.”

### Round 2

The Round 2 questionnaire was developed based on emergent themes from the Round 1 questionnaire and included 49 guideline statements in four domains: recruitment (12 items), casting (22 items), case modifications (5 items), and support (10 items).

#### Recruitment

Participants reported nearly unanimous agreement (> 90%) with all guideline statements. However, they cautioned that “institutional trust is necessary” for educational organizations to recruit prospective SPs, particularly in light of the current sociopolitical climate. They acknowledged that while healthcare providers have trusted relationships with prospective SPs, only passive recruitment methods, such as posted flyers, are acceptable to avoid coercion and compromising clinical care by distracting from “sacred provider time.” This item was revised for inclusion in the Round 3 questionnaire.

#### Casting

Participant agreement with guideline items was largely consistent with emergent themes in open-ended responses from Round 1.

##### Transgender Men and Women

We will focus on casting decisions for simulated encounters of transgender women, noting parallel themes for transgender men. Most participants supported casting transgender women as SPs for transgender women patients (94.3%). Dissenting responses emphasized that matched gender identity does not replace the need for case-specific consent, however, particularly in scenarios where “the portrayed patient is experiencing discrimination.” Participants preferred transfeminine individuals as the best alternative portrayal, followed by AFAB non-binary individuals. While most participants agreed that cisgender women could portray transgender women under limited circumstances, a sizeable minority (25%) strongly recommended against this practice under any circumstance. This dissenting group cited the practice as “[dis]respectful to the lived experience” of TGD individuals and “a disservice to the medical professional you’re trying to train.” Participants almost unanimously agreed that cisgender men should never portray transgender women, consistent with Round 1 results.

Based on participant feedback, we combined items describing the casting of cisgender SPs to portray TGD patients, applying narrow restrictions such as the availability of more appropriate casting options and the case context being unrelated to gender care.

##### Non-binary Individuals

Participants confirmed high acceptability for casting non-binary, bigender, and agender individuals, as well as transgender men and women, as TGD SPs. They recommended limiting cisgender portrayals based on case content and availability of more suitable SP candidates. Participants also confirmed that casting Two-Spirit individuals should be restricted to those who hold this gender identity.

##### Cisgender Portrayal

For the prohibition of casting TGD SPs as explicitly cisgender patients, participants reported borderline agreement (63.2%). Most dissenting responses supported allowing this practice if the SP was comfortable, arguing they likely have lived experience with male and/or female gender identities and to reflect equity in bidirectional representation. We eliminated this item from Round 3 due to borderline acceptability and a literature review revealing simulated patients are almost never explicitly identified as cisgender.^18,19^

#### Case Modifications

Participants reported nearly unanimous agreement (> 90%) with all guideline statements. Three respondents noted that simulation manikins typically exhibit binary gender expressions, which may not align with the SP’s gender identity, suggesting the need to consider auditory and visual components thoughtfully. Two respondents cautioned against including simulated patient’s pronouns in videoconferencing software if the TGD SP is uncomfortable with their inclusion.

#### Support

Participants reported nearly unanimous agreement (> 90%) with nine of the ten guideline statements. However, the statement about disclosing to simulation participants that SPs are from the TGD community received only 73.7% agreement. Concerns included safety, SP consent, and the belief that such disclosure might diminish the simulation’s value as trainees may not have this information in real-world scenarios. As one participant reflected, “I think [trainees] would be more likely to react honestly if they don’t know [an SP] is trans.” Due to these concerns and potential issues for contracted employees, this item was removed from the tentative guidelines. Participants also suggested expanding SPs’ agency to remove themselves from simulations for safety purposes, and this item was revised for inclusion in the Round 3 questionnaire.

### Round 3

The Round 3 questionnaire included 8 revised guideline statements in three domains: recruitment (1 item), casting (6 items), and support (1 item). All guideline statements received > 60% agreement by participants.

#### Recruitment

95.2% of participants agreed with the revised item on passive recruitment techniques by trusted healthcare providers. Dissenting responses were qualitatively compatible with the guideline statement.

#### Casting

Participants’ quantitative agreement with the revised guideline statements regarding transgender women and men was similar to the previous iterations in Round 2. Qualitative responses also remained unchanged. We elected to preserve the revised statements for parsimony as they were in a collapsed form compared to Round 2. Importantly, a stable minority (25%) continued to recommend against cisgender SPs portraying transgender patients under any circumstances, citing the lack of lived experience necessary for appropriate reactions during encounters and the potential offensiveness to the TGD community.

Respondents expressed more favorable agreement with the revised statement on casting transgender men and women to portray non-binary simulated patients, noting that this casting is more appropriate if a non-binary SP is not available.

#### Support

Respondents reported 97.6% agreement with the revised item enhancing the agency of TGD SPs to end a simulation encounter for psychological safety. The sole dissenting response emphasized the importance of SPs fulfilling their contractual obligations.

### Final Guideline

The final guideline consisted of 44 statements across four domains: recruitment (11 items), casting (19 items), case modification (5 items), and support (9 items). Thirty-nine statements were strong recommendations, and five were conditional (Table 2). In the sensitivity analysis, 38 of the 39 strong recommendations retained their classification, while one was downgraded to conditional. Of the five conditional recommendations, one remained, and four were excluded due to agreement rates falling marginally below the consensus threshold of 60% (range 57.3–59.3%). Excluded items shared a common theme, addressing conditional support for casting transgender individuals with discordant sex assigned at birth or cisgender individuals with matched gender identity, as well as one item related to cisgender portrayal of a non-binary individual.

**Table 2:** Extent of Participant Agreement with Final Guideline Statements

Guideline statement	Agree (*N*=43)
**Recruitment best practices**	
*A medical simulation program should ensure the following with respect to hiring and employment:*	
The program should cultivate a positive relationship with the local TGD community.*	100%
Hiring processes should be affirming, including collection of chosen names and pronouns.*	100%
TGD standardized patients should be compensated monetarily for their work.*	94.3%
The program should communicate with prospective actors the non-monetary benefits of being a standardized patient, including personal benefits and contributing to the education of trainees to provide affirming care.*	97.1%
The program should acknowledge the challenges of working as a standardized patient portraying TGD identities, including emotional labor.*	100%
*A medical simulation program may use the following sources to ethically identify TGD community members who may be interested in working as standardized patients:*	
Healthcare professionals who have established trust with the LGBTQ+ community may post the opportunity (i.e., a flyer) in their practice space.*	95.2%
Community organizations focused on LGBTQ+ community needs*	97.1%
Institutional organizations focused on LGBTQ+ community needs*	94.3%
Grassroots and/or support groups for the LGBTQ+ community*	97.1%
Social media spaces that center the needs of the LGBTQ+ community*	97.1%
Community events that center the needs of the LGBTQ+ community*	100%
**Casting of specific gender identities using medical simulation**	
The actor portraying a simulated patient must feel comfortable and give their consent to represent the simulated patient's gender identity.*	97.1%
*Portrayal of a simulated patient who identifies as a transgender woman*	
It is always appropriate for a transgender woman to portray a transgender woman.*	94.3%
It is usually appropriate for a transfeminine individual to portray a transgender woman.*	100%
It is sometimes appropriate for an AFAB individual who identifies as non-binary or genderqueer to portray a transgender woman if a transgender woman or transfeminine individual is not available.^†^	69.0%
It is rarely appropriate for a cisgender woman to portray a transgender woman and only in the following circumstance: if a transfeminine individual is not available, and the case scenario is unrelated to gender-affirming care.^†^	66.7%
It is never appropriate for a transgender man to portray a transgender woman.*	94.3%
It is never appropriate for a cisgender man to portray a transgender woman.*	94.3%
*Portrayal of a simulated patient who identifies as a transgender man*	
It is always appropriate for a transgender man to portray a transgender man.*	91.4%
It is usually appropriate for a transmasculine individual to portray a transgender man.*	97.1%
It is sometimes appropriate for an AMAB individual who identifies as non-binary or genderqueer to portray a transgender man if a transgender man or transmasculine individual is not available.^†^	66.7%
It is rarely appropriate for a cisgender man to portray a transgender man and only in the following circumstance: if a transmasculine individual is not available, and the case scenario is unrelated to gender-affirming care.^†^	69.0%
It is never appropriate for a transgender woman to portray a transgender man.*	94.3%
It is never appropriate for a cisgender woman to portray a transgender man.*	94.3%
*Portrayal of a simulated patient who identifies as non-binary*	
It is always appropriate for a non-binary, genderqueer, or genderfluid individual to portray a non-binary individual.*	94.7%
It is usually appropriate for a bigender or agender individual to portray a non-binary individual.*	97.4%
It is sometimes appropriate for a transgender man or transgender woman to portray a non-binary individual if a non-binary individual is not available.*	81.0%
It is rarely appropriate for a cisgender individual to portray a non-binary individual and only in the following circumstance: if a non-binary individual is not available, and the case scenario is unrelated to gender-affirming care.^†^	71.4%
*Portrayal of a simulated patient who identifies as Two-Spirit*	
It is always appropriate for a Two-Spirit individual to portray a Two-Spirit individual.*	97.4%
It is never appropriate for an individual who does not identify as Two-Spirit to portray a Two-Spirit individual.*	94.7%
**Case modifications in portrayal**	
The use of a manikin does not change who can appropriately portray a TGD simulated patient.*	92.1%
TGD standardized patients should not be asked to alter the acoustic quality of their voice when voicing a manikin.*	100%
The use of teleconferencing software to conduct a simulated patient visit does not change who can appropriately portray a TGD simulated patient.*	94.7%
A simulated patient’s chosen name and pronouns should be displayed in the teleconferencing software.*	94.7%
TGD standardized patients may disable video as needed during a simulated telemedicine visit.*	94.7%
**Training and support of TGD standardized patients**	
*Before any simulation experience*	
Learners should receive diversity and sensitivity training about TGD individuals before joining a simulation event addressing TGD healthcare.*	94.7%
Institutions should obtain informed consent from TGD standardized patients, clearly outlining the nature of the simulation and the potential emotional impact.*	97.4%
*During any simulation experience*	
A TGD standardized patient may end a simulation if they are unable or do not wish to continue.*	97.6%
TGD standardized patients will receive the promise of immediate intervention if they become distressed by a situation without being ignored.*	100%
The simulation program will address distressing situations and will not ignore blatant discrimination.*	100%
A TGD standardized patient should be provided with optional, additional break time between encounters with learners.*	97.4%
A TGD standardized patient should be able to bring a trusted friend or knowledgeable companion to accompany them in medical simulation scenarios.*	100%
*After any simulation experience*	
After simulation events, a debrief session should be organized for TGD SPs to discuss their experiences and receive support.*	100%
A simulation program should establish a robust mechanism for TGD SPs to provide feedback to ensure the simulation experience is affirming.*	100%

^*^Strong recommendation (> 75% agreement)

^†^Conditional recommendation (60–74% agreement)

## DISCUSSION

This study demonstrates the feasibility of collaborating with a local TGD community to develop a preliminary guideline on supporting TGD SPs in medical simulation. It provides specific recommendations on how to ethically recruit, cast, and support TGD SPs, balancing the emotional labor of this work with the educational objectives. Participants reached consensus on 44 recommendations across multiple domains, with the majority classified as strong, and most were robust to sensitivity analysis.

Participants reached consensus on key questions regarding best practices for TGD SPs. A gradient of acceptable casting decisions based on gender identity can guide simulation programs in matching TGD SPs to appropriate cases. Participants suggested limiting cisgender SPs to roles based on gender identity rather than sex assigned at birth and unrelated to gender care. These guidelines aim to broaden the eligible pool of SPs and inform simulation case design. For cases portraying TGD patients, incorporating variations for at least four gender identities—transgender woman, transgender man, AMAB non-binary individual, and AFAB non-binary individual—will ensure a diverse candidate SP pool.

Our findings align with previous qualitative studies of TGD SPs and TGD healthcare providers.^10,11^ A focus group of TGD SPs highlighted similar themes: the importance of lived experience for authentic portrayal, the emotional labor involved, including microaggressions and reliving traumatic healthcare experiences, and the critical role of simulation staff support. Participants advocated for breaks, the ability to end encounters, and post-session debriefings. They also recognized non-monetary benefits, such as improving provider healthcare competency and supporting the TGD community.^10^ Moreover, our findings align with themes from a study of educators in diversity, equity, and inclusion simulation programming for broader minoritized populations, including efforts to minimize re-traumatization and address recruitment challenges due to systemic barriers.^20^

This study has several limitations. This was a single-center study with a small sample size of geographically restricted, mostly AMAB participants who were overwhelmingly young, although age demographics mirrored population-based studies of TGD individuals in the USA.^12^ A plurality of participants identified as non-binary, and only one participant identified as Two-Spirit. Although racial data were not collected, the sample likely comprised mostly white individuals, consistent with the demographics of Middle Tennessee. The sampling strategy may have overrepresented TGD individuals associated with a tertiary academic medical center, potentially leading to a greater acceptance of educational concessions than might be found in a broader community population. Finally, while these guidelines focus on supporting the psychological safety of TGD SPs, we did not examine how they might affect the learning environment for trainees, who also require psychological safety to make mistakes and grow.

Our study offers a framework of best-practice recommendations for supporting TGD SPs, developed with input from the local TGD community. Validating this preliminary guideline with diverse TGD populations, particularly those with intersecting marginalized identities, is essential. This validation will help establish comprehensive best practices in TGD simulation, guiding medical simulation programs in designing and implementing TGD experiences while ensuring a minimum standard of support. Additionally, methodologies like focus groups that allow real-time feedback may be more effective in developing nuanced guidance on cisgender portrayal specifically as these items were not robust to participant attrition in sensitivity analysis.

Incorporating these best practices into TGD healthcare simulations has the potential to enhance the educational value for medical trainees while ensuring the psychological safety of TGD SPs. This approach could foster a new generation of culturally competent physicians, addressing the stigmatizing healthcare environments of the past and helping to mitigate health inequities for a vulnerable population.

## Supplementary Information

Below is the link to the electronic supplementary material.

Supplementary file1 (DOCX 30 KB)

## Data Availability

The data that support the findings of this study are not openly available due to reasons of sensitivity and are available from the corresponding author upon reasonable request.

## References

[1] **Herman JL, Flores AR, O’Neill KK.** How many adults and youth identify as transgender in the United States? The Williams Institute, UCLA School of Law. 2022.

[2] **James SE, Herman JL, Rankin S, Keisling M, Mottet L, Anafi M.** The report of the 2015 U.S. transgender survey. Washington, DC: National Center for Transgender Equality. 2016.

[3] Institute of Medicine. The health of lesbian, gay, bisexual, and transgender people: building a foundation for better understanding. Washington, DC: The National Academies Press. 2011. 10.17226/13128. 22013611

[4] **Chen D, Berona J, Chan Y, et al.** Psychosocial functioning in transgender youth after 2 years of hormones. N Engl J Med. 2023;388(3):240-50. 10.1056/nejmoa2206297. 36652355 10.1056/NEJMoa2206297 PMC10081536

[5] **Baker KE, Wilson LM, Sharma R, Dukhanin V, McArthur K, Robinson KA.** Hormone therapy, mental health, and quality of life among transgender people: a systematic review. J Endocr Soc. 2021;5(4). 10.1210/jendso/bvab011. 10.1210/jendso/bvab011 PMC7894249 33644622

[6] **Streed CG, Michals A, Quinn E, et al.** Sexual and gender minority content in undergraduate medical education in the United States and Canada: current state and changes since 2011. BMC Med Educ. 2024;24:482. 10.1186/s12909-024-05469-0. 38693525 10.1186/s12909-024-05469-0 PMC11064371

[7] **Karpel HC, Sampson A, Charifson M, Zhao D, Kumar V, Sampson MA.** Assessing medical students' attitudes and knowledge regarding LGBTQ health needs across the United States. J Prim Care Community Health. 2023;14:21501319231186729. 10.1177/21501319231186729. 10.1177/21501319231186729 PMC10350786 37449447

[8] **Bohnert CA, Combs RM, Noonan EJ, Weathers AE, Weingartner LA.** Gender minorities in simulation: a mixed methods study of medical school standardized patient programs in the United States and Canada. Simul Healthc. 2021;16(6). 10.1097/SIH.0000000000000532. 10.1097/SIH.0000000000000532 33273422

[9] **Petrey LN, Noonan EJ, Weingartner LA.** Gender diverse representation in patient simulation: a scoping review. Acad Med. 2022;97(11S). 10.1097/ACM.0000000000004926. 10.1097/ACM.0000000000004926 35947464

[10] **Noonan EJ, Weingartner LA, Combs RM, Bohnert C.** Perspectives of transgender and genderqueer standardized patients. Teach Learn Med. 2021;33(2):116-28. 10.1080/10401334.2020.1811096. 32894026 10.1080/10401334.2020.1811096

[11] **Noonan EJ, Combs R, Bohnert C, Decker HR, Black C, Weingartner LA.** Perspectives of transgender and nonbinary health care providers on gender minority patient simulation. Acad Med. 2022;97(11S). 10.1097/ACM.0000000000004873.

[12] **Humphrey-Murto S, Varpio L, Wood TJ.** The use of the Delphi and other consensus group methods in medical education research: a review. Acad Med. 2017;92(10):1491-8. 10.1097/ACM.0000000000001812. 28678098 10.1097/ACM.0000000000001812

[13] **Harris PA, Taylor R, Thielke R, Payne J, Gonzalez N, Conde JG.** Research electronic data capture (REDCap)–a metadata-driven methodology and workflow process for providing translational research informatics support. J Biomed Inform. 2009;42(2):377-81. 10.1016/j.jbi.2008.08.010. 18929686 10.1016/j.jbi.2008.08.010 PMC2700030

[14] **Harris PA, Taylor R, Minor BL, et al.** The REDCap consortium: building an international community of software platform partners. J Biomed Inform. 2019;95:103208. 10.1016/j.jbi.2019.103208. 31078660 10.1016/j.jbi.2019.103208 PMC7254481

[15] **Bartoletta KM, Starr SR.** Health Systems Science. Adv Pediatr. 2021;68:1-19. 10.1016/j.yapd.2021.05.001 34243847 10.1016/j.yapd.2021.05.001 PMC9188469

[16] **Diamond IR, Grant RC, Feldman BM, et al.** Defining consensus: a systematic review recommends methodologic criteria for reporting of Delphi studies. J Clin Epidemiol. 2014;67(4):401-9. 10.1016/j.jclinepi.2013.12.002. 24581294 10.1016/j.jclinepi.2013.12.002

[17] **Wall CSJ, Patev AJ, Benotsch EG.** Trans broken arm syndrome: A mixed-methods exploration of gender-related medical misattribution and invasive questioning. Soc Sci Med. 2023;320:115748. 10.1016/j.socscimed.2023.115748 36736052 10.1016/j.socscimed.2023.115748

[18] **Bod J, Buck S, Chandler I, et al.** LGBTQ+ individuals are not explicitly represented in emergency medicine simulation curricula. MedEdPublish 2024, 14:30. 10.12688/mep.20242.1. 38932993 10.12688/mep.20242.1 PMC11200058

[19] **Bod J.** LGBTQ+ Individuals are not explicitly represented in emergency medicine simulation curricula. Published online 2024. 10.7910/DVN/KY4TF0. 10.12688/mep.20242.1 PMC11200058 38932993

[20] **Sibbald M, Last N, Keuhl A, Marcotte E, Benjamin C.** Challenges facing standardised patients representing equity-deserving groups: insights from health care educators. Med Educ. 2023;57(6):516-22. 10.1111/medu.15085. 36987681 10.1111/medu.15085

