# Supplementary material for: Community-Engaged Qualitative Study on Supporting Transgender and Gender-Diverse Standardized Patients in Medical Simulation
Source: J Gen Intern Med. 2025 Jun 24;40(13):3137–45. doi: 10.1007/s11606-025-09640-1 (PMC12508243; doi:10.1007/s11606-025-09640-1)
Supplement: Supplementary file 1 — Supplementary file1 (DOCX 30 KB) [file 11606_2025_9640_MOESM1_ESM.docx]

**Appendices**

Supplementary Table 1 – Hierarchical Codebook for Round 1 Participant Responses

| **Number** | **Category** | **Definition** | **Rule** |
| --- | --- | --- | --- |
| 1 | Identity | Use this to specify the gender identity that is referred to in the quote you are coding. | If no identify is being referred to in the quote, then do not use a category 1 code |
| 1.1 | Transgender and gender-diverse | Discordance between sex assigned at birth and gender identity |  |
| 1.1.1 | Fixed identities | This set of gender identities tends to be fixed |  |
| 1.1.1.1 | Transgender woman | Transgender woman |  |
| 1.1.1.2 | Transgender man | Transgender man |  |
| 1.1.1.3 | Bigender | Bigender |  |
| 1.1.1.4 | Agender | Agender |  |
| 1.1.2 | Fluid | This set of gender identities tends to be fluid |  |
| 1.1.2.1 | Demigender | Demigender |  |
| 1.1.2.2 | Non-binary | Non-binary |  |
| 1.1.2.3 | Genderqueer | Genderqueer |  |
| 1.1.2.4 | Genderfluid | Genderfluid |  |
| 1.1.2.5 | Genderqueer | Genderqueer |  |
| 1.2 | Two Spirit | Two Spirit |  |
| 1.3 | Cisgender | Concordance between sex assigned at birth and gender identity |  |
| 1.3.1 | Cisgender woman | Cisgender woman |  |
| 1.3.2 | Cisgender man | Cisgender man |  |
| 1.4 | Unable to identify or not specific | Use this if the respondent is referring to a person with or portraying a gender identity, but the statement is not specific enough to code |  |
| 2 | Role | the Role being referred to in the quote | If the statement includes both, use both codes (e.g., a cisgender man cannot portray a transgender man) |
| 2.1 | Patient | The respondent is talking about the gender identity of the patient being portrayed |  |
| 2.2 | Actor | The respondent is talking about the gender identity of the actor |  |
| 3 | Essential Element | The respondent talks about the characteristics or conditions that are necessary for a sensitive portrayal by a standardized patient |  |
| 3.1 | Respect | Respect can be at the individual (the actor respects) or institutional (the institution has respect) |  |
| 3.1.1 | Individual | The actor has respect or is affirming of gender diversity |  |
| 3.1.2 | Institutional | The institution has respect or is affirming of gender diversity |  |
| 3.2 | Lived experience | Some aspect of the life experience of an actor makes it possible to portray a specific identity |  |
| 3.3 | Emotional identification | The actor has empathy, sensitivity, and/or understanding of the emotional experiences of the identify being portrayed |  |
| 3.4 | Fixed or fluid | A distinction is being made between an actor who clearly understands how having a fixed and fluid identity differs |  |
| 3.5 | Cultural competence/intersectionality | When experience with a specific cultural identity is relevant, or when a cultural characteristic intersects with gender identity |  |
| 3.6 | Internal/external | Statement about the congruence or incongruence between how someone feels and how they present themselves publicly |  |
| 3.7 | Visual cues | Use of visual cues to portray and identity is referenced |  |
| 3.8 | Auditory cues | Use of sound or voice cues to portray an identify is referenced |  |
| 3.9 | Clinical context | The nature of the health condition or clinically relevant variables in included in the response |  |
| 3.10 | Disclosure/expectations | The need to set expectations with the medical students regarding the portrayal of a gender minority case |  |
| 3.11 | Other characteristic | Some other characteristic, condition, or qualification is identified |  |
| 4 | Qualification | when a clear statement of who may or may not qualify to portray a specific gender identity |  |
| 4.1 | Qualifies to portray | The characteristic or experience qualifies an actor to portray an identity |  |
| 4.2 | Does not qualify to portray | The characteristic or experience disqualifies an actor to portray an identity |  |
| 5 | Training/Preparation | Statements about how to train or prepare an actor to serve as a standardized patient |  |
| 5.1 | Clinical | Training the actor to be knowledgeable about clinical conditions, symptoms, complications, etc. |  |
| 5.2 | Sensitivity/Diversity | Training that educates the actor on gender identities, lived experiences, cultural context, or other information that would improve sensitivity and accuracy |  |
| 5.3 | Appropriateness | Clear guidelines to use appropriate and avoid inappropriate behaviors that might offend others or fail to portray and identify |  |
| 5.4 | Subtle Cues | Training on using subtle cues to portray an identity when the identification is to be inferred not announced during the simulation |  |
| 5.5 | Other training | Some other aspect of training is identified |  |
| 6 | Setting factors | The discussion refers to the setting in which the portrayal is to occur |  |
| 6.1 | Modality | In person, teleconference, other thoughts around modality of portrayal |  |
| 6.2 | Case content | Details of clinical conditions as part of the context |  |
| 6.3 | Manikin | Use of a manikin or mechanical simulation |  |
| 6.4 | Psychological safety | Statements reflecting ability to safely express self without psychological harm |  |
| 6.5 | Physical safety | Statements reflecting ability to safely express self without physical harm |  |
| 6.6 | Other setting factors | Some other aspect of the setting or context of the simulation is identified |  |
| 7 | Recruitment factors | Statements about how to recruit standardized patients from the TGD community |  |
| 7.1 | Sources | Recruitment sources for potential standardized patients |  |
| 7.1.1 | Lay Individuals | Specific individuals in the community who can recruit standardized patients |  |
| 7.1.2 | Professionals | Trusted individuals who hold expertise can recruit standardized patients |  |
| 7.1.3 | Community organizations | LGBTQ+-focused community groups |  |
| 7.1.4 | Institutional organizations | LGBTQ+-focused institutional bodies |  |
| 7.1.5 | Events | Specific events which garner attendance from TGD community |  |
| 7.1.6 | Grassroots/support organizations | Loosely affiliated LGBTQ-focused support groups |  |
| 7.2 | Barriers | Statement about a specific barrier to recruitment |  |
| 7.3 | HR/employment processes | Statements about institutional hiring and employment |  |
| 7.4 | Perceived benefits | Putative monetary and non-monetary benefits of being a standardized patient |  |
| 7.5 | Other recruitment factors | Some other aspect of recruitment is identified |  |
